# Evaluation of amyloid A and haptoglobin in the serum of cats with respiratory diseases

**DOI:** 10.3389/fvets.2026.1798605

**Published:** 2026-04-23

**Authors:** Hannah Gareis, Bianka Schulz

**Affiliations:** Clinic of Small Animal Medicine, Ludwig-Maximilians-University of Munich, Munich, Germany

**Keywords:** acute phase proteins, airways, heart failure, infection, neoplasia, rhinitis

## Abstract

**Background:**

Acute-phase proteins (APPs) change in concentration during systemic inflammation.

**Objectives:**

To investigate serum concentrations of amyloid A (SAA) and haptoglobin (Hp) in various respiratory diseases in cats, and assess their potential to distinguish between different respiratory conditions.

**Material and methods:**

Serum samples of 102 cats with respiratory signs and 20 healthy control cats were prospectively collected and tested for SAA and Hp. Cats were grouped based on conditions into feline lower airway disease (FLAD, *n* = 26), infectious lower airway or pleural space disease (*n* = 12), neoplastic lower airway or pleural space disease (*n* = 17), congestive heart failure (CHF, *n* = 29) and upper airway disease (UAD, *n* = 18).

**Results:**

Median SAA concentrations were significantly higher in cats with infectious lower airway or pleural space disease (2.61 μg/mL, IQR: 0.57–15.45 μg/mL) compared to cats with FLAD (0.14 μg/mL, IQR: 0.10–0.63 μg/mL; *p* < 0.001). However, median values in all groups were within the reference range (< 6.7 μg/mL) and did not significantly differ from healthy controls. Median Hp levels were significantly higher in cats with UAD (2.41 g/L, IQR: 2.21–2.87 g/L; *p* = 0.001), neoplastic (2.45 g/L, IQR: 2.10–2.86 g/L; *p* < 0.001) and infectious lower airway or pleural space disease (2.60 g/L, IQR: 2.40–2.67 g/L; *p* < 0.001) compared with FLAD (1.76 g/L, IQR: 0.99–2.26 g/L). Cats with UAD (*p* < 0.001), neoplasia (*p* < 0.001), infectious lower airway or pleural space disease (p < 0.001) and CHF (2.31 g/L, IQR: 0.84–2.57 g/L; *p* = 0.002) showed significantly higher Hp values compared to healthy cats (1.25 g/L, IQR: 0.81–1.83 g/L) (reference range < 1.9 g/L).

**Conclusion:**

SAA and Hp are not suitable for clearly differentiating between different respiratory conditions in cats. However, increased SAA levels are more indicative of infections of the lower airways or pleural space than of FLAD, which could be helpful for therapeutic considerations.

## Introduction

Acute-phase proteins (APPs) are known to be a heterogeneous group of proteins synthesized in the liver, which change in serum concentration as a result of inflammation. This is part of the specific immune defenses of the body—known as acute-phase response—against inflammatory stimuli triggered by infections, trauma, immune reactions and neoplastic diseases (1). APPs can therefore be used to evaluate the body’s response to systemic inflammatory disease and also play an important role in therapeutic monitoring of various diseases (2).

In cats, the most studied APPs include serum amyloid A (SAA), the major feline APP, as well as haptoglobin (Hp) and alpha glycoprotein (AGP), which are considered moderate APPs (2, 3). The difference between major and moderate APPs lies in the magnitude and speed of the increase after stimulation: While major APPs can increase dramatically > 1,000-foldwith a peak at 24–48 h, moderate APPs increase 5- to 10-fold on activation with a peak after 2–3 days (2).

So far, APPs have been studied in cats with various diseases, including infectious, endocrine, liver and gastrointestinal diseases, heart disease, neoplasia and sepsis (4–15).

However, previous studies investigating APPs in cats showed controversial results with regard to levels measured in cats with different diseases: one study revealed no increase in APPs in cats with diabetes mellitus, cardiomyopathy and hyperthyroidism compared to healthy cats (14), while other studies showed an increase in APP concentration in these diseases (4, 16, 17).

In dogs, studies have been carried out in patients with different respiratory diseases investigating C-reactive protein (CRP) as the major canine APP. These have shown that CRP is significantly higher in dogs with bacterial pneumonia than in other respiratory diseases and congestive heart failure (CHF) (18). In addition, CRP can be used to monitor the efficacy of antibiotic treatment in dogs with bacterial pneumonia (18, 19).

To date, there has been only little research investigating APPs in cats with respiratory disease. A recent study has shown that cats with CHF had significantly higher SAA than cats with compensated cardiomyopathy and healthy cats (16). Another study showed that cats with pneumonia and upper respiratory tract infections had higher SAA values compared to healthy cats (14).

Studies investigating APPs in different feline respiratory diseases are lacking so far, and whether APPs are suitable for differentiating between various feline respiratory conditions is currently unclear. In daily clinical practice, veterinarians are frequently faced with the challenge of distinguishing between infectious and non-infectious respiratory diseases in cats. This differentiation is of particular importance, as the therapeutic approaches for these conditions can differ substantially. However, clinical signs are often overlapping and non-specific, and advanced diagnostic procedures are not always feasible.

The objective of this study was therefore to investigate serum concentrations of SAA and Hp in various respiratory diseases in cats, and to determine whether these APPs could be used as markers to distinguish between different respiratory conditions. A second aim was to evaluate the association between levels of SAA and Hp and clinical signs in cats with various respiratory diseases. We hypothesized that cats with infectious lower airway or pleural space disease would have significantly higher levels of SAA and Hp in comparison to cats with other respiratory diseases.

## Materials and methods

The study was approved by the Ethics Committee of the Centre for Clinical Veterinary Medicine of the Ludwig Maximilian University (LMU) in Munich (No. 410–07-08-2024).

### Study population

Serum samples from 102 cats that had been presented to the LMU Small Animal Clinic due to respiratory clinical signs for emergency or regular work-up, and serum samples of 20 clinically healthy cats were tested for SAA- and Hp. Serum used for the analysis was collected at the time of the cats’ initial presentation prior to the initiation of therapy. Serum samples were centrifuged and stored at −80 °C. All serum samples of diseased and healthy cats were sent collectively to the external laboratory (LABOKLIN Laboratory for Clinical Diagnostics GmbH & Co. KG), respectively. SAA and Hp were tested via photometric determination. SAA was measured using an immunoturbidimetric assay, whereas Hp was determined by a colorimetric method based on a peroxidase reaction. The reference interval established by the laboratory for SAA is < 6.7 μg/mL and for Hp < 1.9 g/L.

Serum from diseased cats was included if cats were diagnosed with an underlying respiratory or cardiac disease. If a cat was presented with tachypnea due to stress or pain (non-cardiac or non-respiratory disease), or without disease classification, the serum was not included in the study. Serum for the healthy control group was included from cats presented for blood donation or health care, which were unremarkable on clinical examination, hematology and serum biochemistry.

Cats were grouped according to documented diseases: upper airway disease (UAD), feline lower airway disease (FLAD), infectious lower airway or pleural space disease, neoplastic disease of the lower airways or pleural space and congestive heart failure (CHF). The various examinations carried out for diagnosis of the underlying disease were performed upon decision of the primary case managing clinician and consisted of clinical examination, blood tests, faecal examinations, imaging (radiography, ultrasound, echocardiography, computed tomography), endoscopic examinations, bronchoalveolar-lavage-fluid (BALF) samples, microbiological tests, cytology and histopathology. Cats were assigned to the respective disease groups only after a definitive diagnosis had been established based on the diagnostic procedures performed. Cats diagnosed with any type of upper airway disease were assigned to the UAD group. For classification into the FLAD group, all recorded findings, including results from BAL had to be consistent with feline asthma or sterile chronic bronchitis. Bacteriological examinations and PCR for *Mycoplasma-ssp.*-PCR in BALF had to be negative. To be assigned to the group of infectious lower airway or pleural disease, the infection had to be confirmed in BALF or pleural effusion samples. Cats with heart failure, based on radiographic and echocardiographic findings, were classified as CHF. Clinical parameters were evaluated based on documented data: respiratory rate at admission, history of coughing, respiratory distress, nasal discharge, anorexia, lethargy and duration of clinical signs before admission. The cats were treated on discretion of the attending veterinarian for their respective diagnosis. Survival was evaluated based on discharge or death related to the inpatient or outpatient stay within the context of the illness. The documented data on group classification and clinical parameters were evaluated by the authors. It should be acknowledged that disease groups comprise heterogeneous disease entities which may introduce variability within groups and should therefore be recognized as a limitation of the study.

### Statistical analysis

The statistical software SPSS version 28.0.1.0 was used for data analysis. To test for parametric distribution, the Shapiro–Wilk test was applied. Data was presented as mean ± SD for normally distributed data, or median and interquartile range (IQR) for non-normally distributed data.

For comparison of SAA and Hp between disease groups, the Kruskal-Wallis test or ANOVA were used, depending on distribution and variance. Subsequently, *p*-values were corrected using the Bonferroni method for multiple comparisons. The comparison of SAA and Hp regarding clinical signs was evaluated by Mann–Whitney-U-test as data was not normally distributed. Correlations were analysed using Kendall rank correlation coefficient *r*. Correlations were considered very weak (*r* = 0.00–0.19), weak (*r* = 0.20–0.39), moderately strong (*r* = 0.40–0.59), strong (*r* = 0.60–0.79) and very strong (*r* = 0.80–1.0). For all tests, the significance level was set at *p* < 0.05.

## Results

### Study population

In the diseased group, a total of 54 male (49 castrated, 5 intact) and 48 female (37 spayed, 11 intact) cats were included. The breeds included were Domestic Shorthair (*n* = 51), British Shorthair (*n* = 11), mixed-breed (*n* = 8), Maine Coon (*n* = 7), Siamese (*n* = 4), Abyssinian (*n* = 3), Norwegian Forest Cat (*n* = 3), Oriental Shorthair (*n* = 3), Persian (*n* = 3), Sphinx (*n* = 2) and one each of the breeds Bengal, British Longhair, Burmese, Domestic Longhair, Half Angora, Thai, and Turkish Van. The median age of the cats was 7 years (IQR: 3–11 years) and the median duration of clinical signs prior to admission was 10 days (IQR: 1–202.5 days).

Eighteen cats (17.6%) were included in the UAD group, 26 cats (25.4%) were grouped in FLAD, 12 cats (11.8%) in infectious lower airway or pleural space disease, 17 cats (16.7%) in neoplastic disease of the lower airways or pleural space and 29 cats (28.4%) in CHF.

Diseases diagnosed in cats with UAD were chronic rhinitis (*n* = 5), upper infectious respiratory disease (*n* = 4), nasal neoplasia (*n* = 4), laryngeal neoplasia (*n* = 2), nasal foreign body (*n* = 1), nasopharyngeal pseudomembrane (*n* = 1) and nasopharyngeal polyp (*n* = 1). Cats in the infectious lower airway or pleural space disease group were diagnosed with bacterial bronchopneumonia (*n* = 6), pyothorax (*n* = 3), *Mycobacterium avium* infection (*n* = 2) and *Aerulostrongylus abstrusus* lungworm infection (*n* = 1).

The duration of clinical signs before presentation differed significantly between the disease groups (*p* < 0.001), with cats with CHF showing the shortest duration compared to the other diseases (Table 1).

**Table 1:** Duration of clinical signs before presentation, age of the cats and respiratory rate at admission in the respective disease group.

Disease group	Duration of clinical signs	Age	Respiratory rate at admission
Feline lower airway disease	365 days (90–639 days)	3.5 years (2–8.5 years)	40/min (32-77/min)
Infectious lower airway or pleural space disease	4.5 days (1–38 days)	4.5 years (2–7.25 years)	70/min (54-84/min)
Neoplastic lower airway or pleural space disease	14 days (5–42 days)	11 years (9–14 years)	60/min (36-80/min)
Congestive heart failure	0 days (0–1 days)	10 years (3–11 years)	72/min (60-80/min)
Upper airway disease	90 days (31–365 days)	7 years (5.25–10 years)	36/min (29-39/min)
Healthy control group	n/a	4.5 years (3–6 years)	53/min (36-80/min)

Data are expressed as medians (IQR).

Respiratory distress was described in 71/102 cats (69.6%) and 37/102 cats (36.3%) had a history of coughing. Sixteen cats (15.7%) showed nasal discharge at presentation, 58 cats (56.9%) were lethargic and 33 cats (32.4%) were anorectic at the time of presentation. The median respiratory rate at presentation was 53/min (IQR: 36/min-80/min). The respiratory rate at presentation was significantly higher in cats with neoplastic (*p* < 0.001) and infectious (*p* < 0.001) disease and in CHF (*p* < 0.001) compared to cats with UAD (Table 1).

The healthy group of cats consisted of 14 male (12 castrated, 2 intact) and 6 female neutered cats of the following breeds: Domestic Shorthair (*n* = 11), Ragdoll (*n* = 3), British Shorthair (*n* = 2), Maine Coon (*n* = 2), Siberian (*n* = 1) and Egyptian Mau (*n* = 1). The median age of the healthy cats was 4.5 years (IQR: 3–6 years). All cats included in the healthy control group were unremarkable in the clinical and laboratory diagnostic examinations performed.

There was a significant difference in the age of cats between the different disease groups (*p* < 0.001; Table 1), whereby cats with neoplastic diseases were older than cats with FLAD (*p* < 0.001), infectious lower airway or pleural space disease (*p* < 0.001) and healthy cats (*p* < 0.001).

### Amyloid A levels in various respiratory diseases

**Figure 1:**
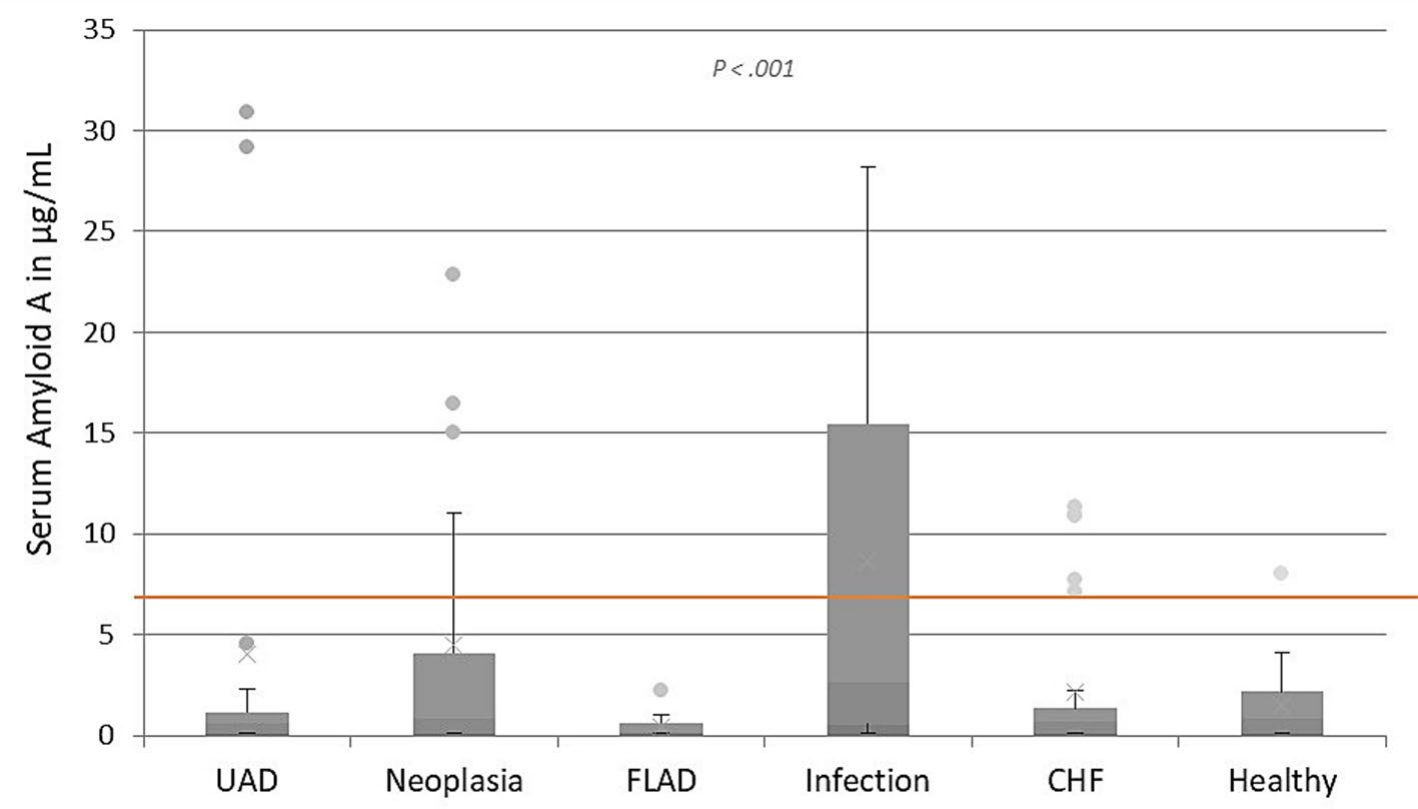
Levels of SAA in μg/mL in cats with upper airway disease (UAD), neoplastic lower airway or pleural space disease, feline lower airway disease (FLAD), infectious lower airway or pleural space disease, CHF, and healthy control cats. The line inside the boxes demonstrates the median SAA value of all the cats included in each group, while the upper and lower boxes show the IQR. The upper and lower whiskers represent the minimum and maximum values. The dots show outliers. The reference range for SAA is < 6.7 μg/mL, indicated by the red line.

The SAA values differed significantly comparing all the different disease groups (*p* = 0.03) and are illustrated in Figure 1. A significant difference was detected between cats diagnosed with FLAD (median: 0.14 μg/mL, IQR: 0.10–0.63 μg/mL) and cats with infectious lower airway or pleural space disease (median: 2.61 μg/mL, IQR: 0.57–15.45 μg/mL), *p* < 0.001. There were no other significant differences between SAA values of the different disease groups and no significant difference found in levels of SAA between healthy cats and diseased cats. The median SAA levels of all disease groups were within the reference range.

In the group of cats with UAD, two cats showed strongly elevated SAA values (29 μg/mL and 30 μg/mL). Histopathologically purulent rhinitis was diagnosed in both cats. All three cats in the neoplastic lower airway or pleural space disease group with an SAA value > 15 μg/mL had pleural effusion secondary to intrathoracic neoplasia. All three cats with pyothorax had increased values for SAA (14 μg/mL, 18 μg/mL, 23 μg/mL) within the group of infectious lower airway or pleural space disease. Of the 6 cats diagnosed with bacterial bronchopneumonia, two cats showed SAA values above the reference range (28 μg/mL and 12 μg/mL). Cats with *Mycobacterium avium* infection and *Aerulostrongylus abstrusus* lungworm infection showed SAA levels within the reference range.

### Haptoglobin levels in various respiratory diseases

**Figure 2:**
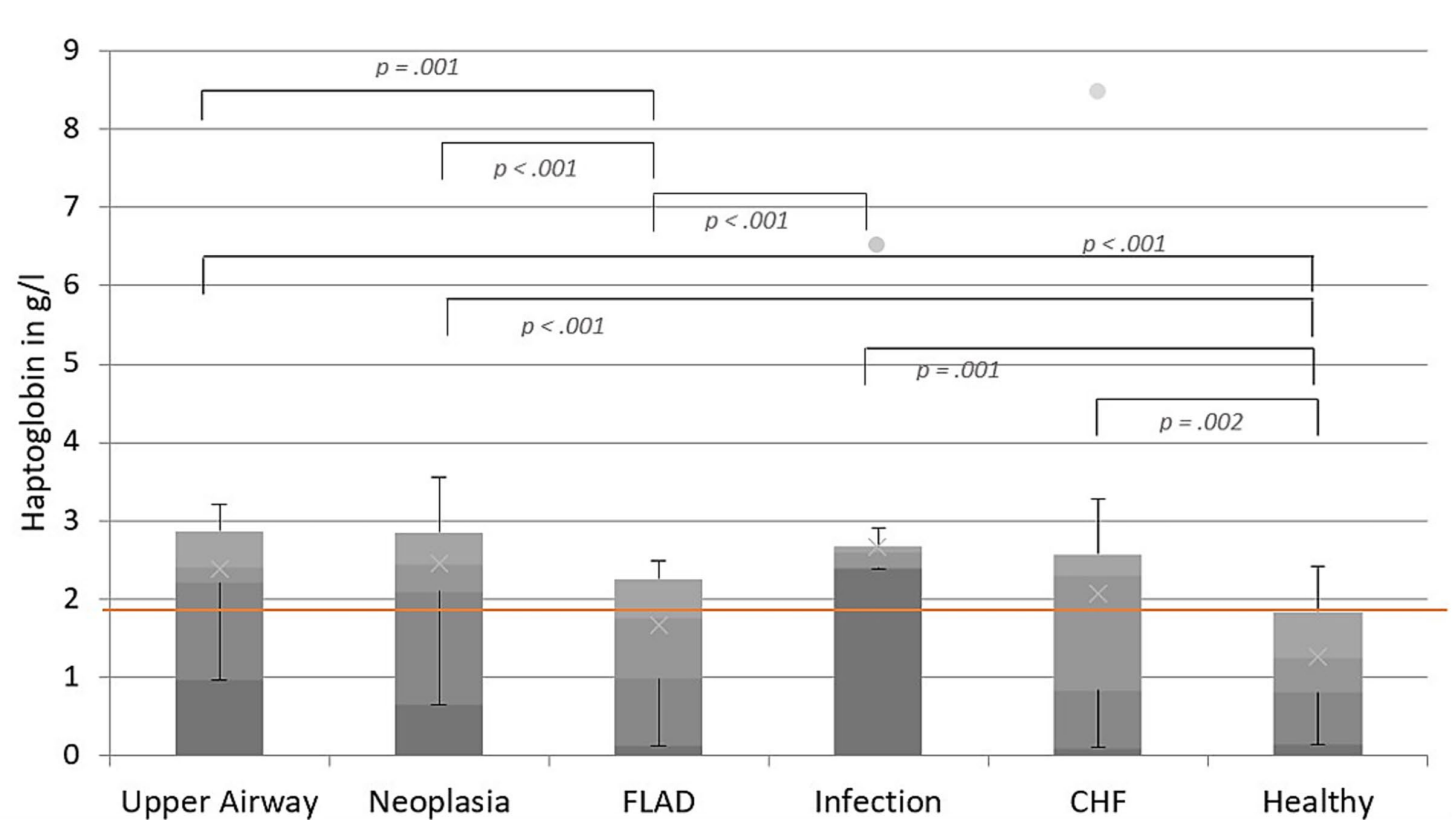
Levels of Hp in g/L in cats with upper airway disease (UAD), neoplastic lower airway or pleural space disease, feline lower airway disease (FLAD), infectious lower airway or pleural space disease, CHF, and healthy control cats. The line inside the boxes demonstrates the median SAA value of all the cats included in each group, while the upper and lower boxes show the IQR. The upper and lower whiskers represent the minimum and maximum values. The dots show outliers. The reference range for Hp is < 1.9 g/L, indicated by the red line.

The levels of Hp in cats with different respiratory diseases showed significant differences when comparing all disease groups (*p* < 0.001): Hp was significantly lower in the FLAD group (median: 1.76 g/L, IQR: 0.99–2.26 g/L) compared to the infectious lower airway or pleural space disease (median: 2.60 g/L, IQR: 2.40–2.67 g/L; *p* < 0.001), neoplastic lower airway or pleural space (median: 2.45 g/L; IQR: 2.10–2.86 g/L; *p* < 0.001) and UAD group (median: 2.41 g/L, IQR: 2.21–2.87 g/L; *p* = 0.001). The Hp levels in cats with UAD (*p* < 0.001), neoplastic lower airway or pleural space disease (*p* < 0.001), infectious lower airway or pleural space disease (*p* < 0.001) and CHF (median: 2.31 g/L, IQR: 0.84–2.57 g/L; *p* = 0.002) were significantly higher compared to the healthy control group (median: 1.25 g/L, IQR: 0.81–1.83 g/L). The Hp levels of all cats are illustrated in Figure 2.

### Amyloid A and haptoglobin levels and signalement

Both in the diseased and healthy control group, there were no significant differences in the evaluation of SAA and Hp levels comparing male and female cats and no correlation detected between SAA or Hp levels and age.

### The association between amyloid A levels and clinical parameters

No significant correlation was found between the respiratory rate at admission and the SAA values in cats with respiratory disease (Table 2). The duration of clinical signs before presentation did not correlate significantly with the level of SAA of all diseased cats either. However, there was a weak positive correlation between the SAA levels of cats with CHF and the duration of disease (*r* = 0.32, *p* = 0.04) and a moderate negative correlation between SAA concentrations of cats with UAD and the duration of clinical signs prior to presentation (*r* = − 0.40, *p* = 0.03) (Table 3).

**Table 2:** Correlation of SAA levels with respiratory rate at admission.

Disease group	Respiratory rate at admission	Amyloid A	Correlation coefficient *r*	*p*-value
Feline lower airway disease	40/min (32-77/min)	0.14 μg/mL (0.10–0.63 μg/mL)	0.17	0.25
Infectious lower airway or pleural space disease	70/min (54-84/min)	2.61 μg/mL (0.57–15.5 μg/mL)	0.24	0.30
Neoplastic lower airway or pleural space disease	60/min (36-80/min)	0.86 μg/mL (0.10–4.10 μg/mL)	0.00	1.00
Congestive heart failure	72/min (60-80/min)	0.70 μg/mL (0.10–1.36 μg/mL)	−0.11	0.45
Upper airway disease	36/min (29-39/min)	0.61 μg/mL (0.10–1.14 μg/mL)	−0.02	0.90
All diseased cats	53/min (36/min-80/min)	0.60 μg/mL (0.10–1.35 μg/mL)	0.11	0.12

Data are expressed as medians (IQR).

**Table 3:** Correlation of SAA levels with duration of clinical signs.

Disease group	Duration of clinical signs	Amyloid A	Correlation coefficient *r*	*P*-value
Feline lower airway disease	365 days (90–639 days)	0.14 μg/mL (0.10–0.63 μg/mL)	−0.26	0.10
Infectious lower airway or pleural space disease	4.5 days (1–38 days)	2.61 μg/mL (0.57–15.5 μg/mL)	−0.36	0.11
Neoplastic lower airway or pleural space disease	14 days (5–42 days)	0.86 μg/mL (0.10–4.10 μg/mL)	0.10	0.58
Congestive heart failure	0 days (0–1 days)	0.70 μg/mL (0.10–1.36 μg/mL)	0.32	**0.04**
Upper airway disease	90 days (31–365 days)	0.61 μg/mL (0.10–1.14 μg/mL)	−0.40	**0.03**
All diseased cats	10 days (1–202.5 days)	0.60 μg/mL (0.10–1.35 μg/mL)	−0.14	0.05

Data are expressed as medians (IQR). Bold values indicate statistical significance.

**Figure 3:**
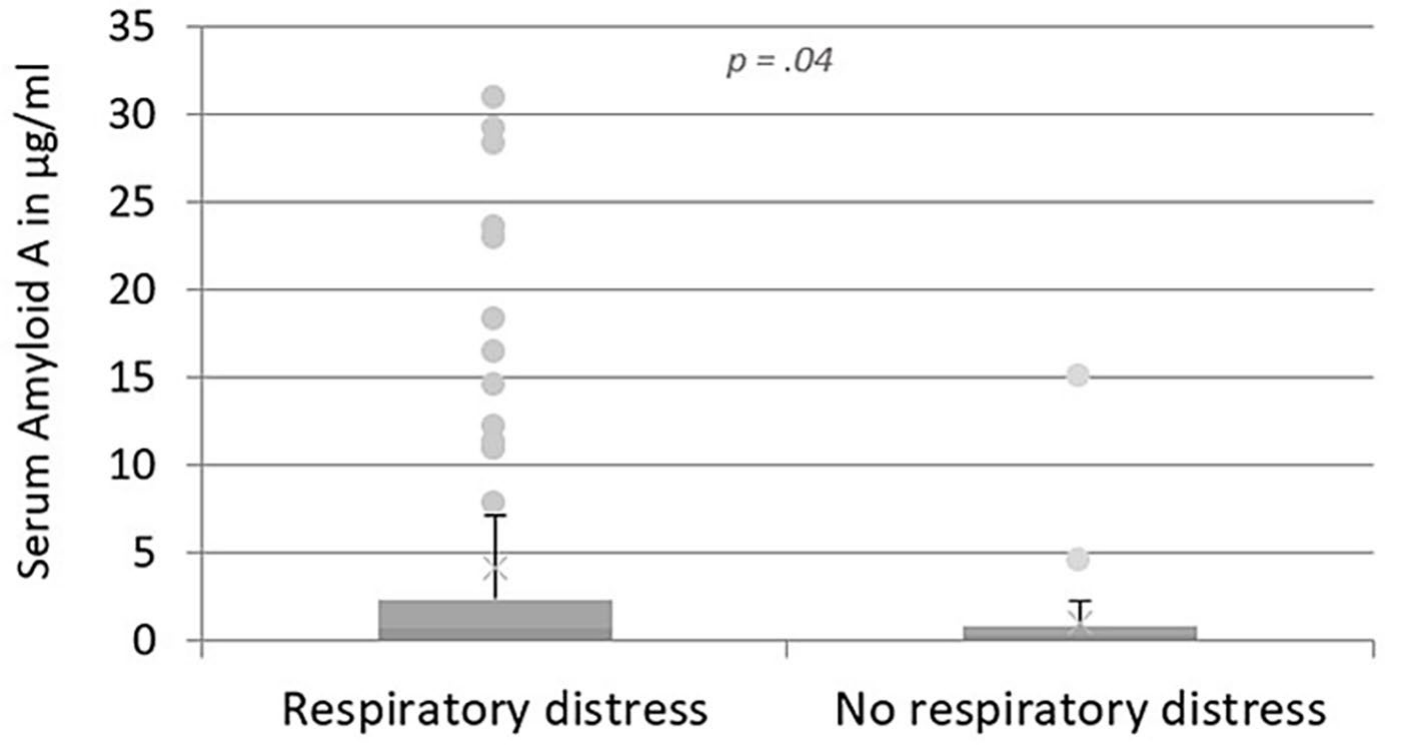
Levels of SAA in μg/mL in cats with respiratory disease, comparing cats with respiratory distress and without respiratory distress. The box plot presents the median SAA value of all the cats with the IQR. The upper and lower whiskers represent the minimum and maximum values. The dots show outliers. Cats with respiratory distress had significantly higher SAA values compared to cats without respiratory distress.

When investigating whether cats with different clinical signs had deviating SAA values, it was shown that cats with respiratory distress had significantly higher SAA values compared to cats without respiratory distress (median: 0.70 μg/mL, IQR: 0.10–2.36 μg/mL versus 0.36 μg/mL, IQR: 0.10–0.87 μg/mL; *p* = 0.04, Figure 3).

**Figure 4:**
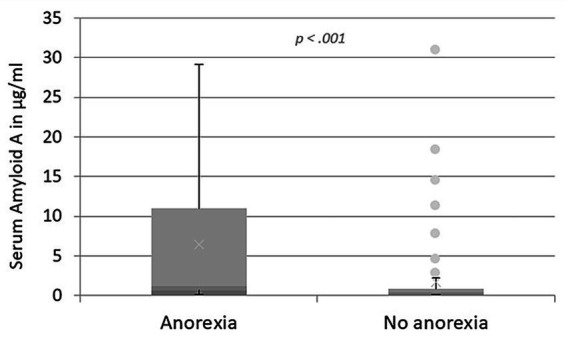
Levels of SAA in μg/mL in cats with respiratory disease, comparing cats with anorexia and cats with normal appetite. The box plot presents the median SAA value of all the cats with the IQR. The upper and lower whiskers represent the minimum and maximum values. The dots show outliers. Cats with anorexia had significantly higher SAA values compared to cats without anorexia.

In addition, cats with anorexia showed significantly higher SAA values compared to cats with no anorexia (median: 1.15 μg/mL, IQR: 0.64–10.98 μg/mL versus 0.39 μg/mL, IQR: 0.10–0.84 μg/mL; *p* < 0.001, Figure 4).

Lethargic cats had a significantly higher median SAA value compared to cats without lethargy (median: 0.76 μg/mL, IQR: 0.10–6.37 μg/mL versus 0.27 μg/mL, IQR: 0.10–0.80 μg/mL; *p* = 0.003). Cats with a history of coughing did not have higher values than cats without a cough.

Despite statistically significant differences, the medians of all groups compared were within the reference range of the laboratory.

### The association between haptoglobin and clinical parameters

No significant correlation was found between the respiratory rate at presentation of cats with respiratory disease and the Hp values (Table 4). The duration of clinical signs before presentation did not correlate significantly with the level of Hp at admission (Table 5). When investigating whether cats with different clinical signs had varying Hp values, it was shown that cats with lethargy (median: 2.45 g/L, IQR: 1.92–2.80 g/L) had a significantly higher Hp value than cats without (median: 2.11 g/L, IQR: 1.26–2.37 g/L) (*p* = 0.002). Cats that were anorexic had significantly higher Hp values (median: 2.48 g/L, IQR: 2.20–2.79 g/L) compared to cats with normal appetite (median: 2.21 g/L, IQR: 1.36–2.45 g/L) (*p* = 0.007). Cats with coughing and respiratory distress did not have higher Hp values than cats that did not show these clinical signs.

**Table 4:** Correlation of Hp levels with respiratory rate at admission presented as median with ICQ.

Disease group	Respiratory rate at admission	Haptoglobin	Correlation coefficient *r*	*p*-value
Feline lower airway disease	40/min (32-77/min)	1.76 g/L (0.99–2.26 g/L)	0.27	0.06
Infectious lower airway disease	70/min (54-84/min)	2.60 g/L (2.41–2.67 g/L)	0.24	0.30
Neoplastic lower airway disease	60/min (36-80/min)	2.45 g/L (2.10–2.85 g/L)	−0.29	0.12
Congestive heart failure	72/min (60-80/min)	2.31 g/L (0.84–2.67 g/L)	0.13	0.35
Upper airway disease	36/min (29-39/min)	2.40 g/L (2.21–2.87 g/L)	0.02	0.91
All diseased cats	53/min (36-80/min)	2.33 g/L (1.57–2.57 g/L)	0.04	0.57

**Table 5:** Correlation of Hp levels with duration of clinical signs presented as median with ICQ.

Disease group	Duration of clinical signs	Haptoglobin	Correlation coefficient *r*	*p*-value
Feline lower airway disease	365 days (90–639 days)	1.76 g/L (0.99–2.26 g/L)	−0.20	0.17
Infectious lower airway or pleural space disease	4.5 days (1–38 days)	2.60 g/L (2.41–2.67 g/L)	−0.25	0.27
Neoplastic lower airway or pleural space disease	14 days (5–42 days)	2.45 g/L (2.10–2.85 g/L)	0.13	0.48
Congestive heart failure	0 days (0–1 days)	2.31 g/L (0.84–2.67 g/L)	0.18	0.24
Upper airway disease	90 days (31–365 days)	2.40 g/L (2.21–2.87 g/L)	0.01	0.94
All diseased cats	10 days (1–202.5 days)	2.33 g/L (1.57–2.57 g/L)	−0.05	0.48

### Short-term survival and death

**Figure 5:**
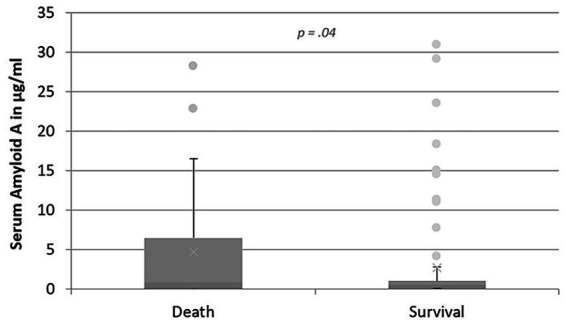
Levels of SAA in μg/mL in cats with respiratory disease, comparing cats that died/were euthanized during hospitalization and cats that were discharged alive. The box plot presents the median SAA value of all the cats with the IQR. The upper and lower whiskers represent the minimum and maximum values. The dots show outliers. Cats that died had significantly higher SAA values compared to survivors.

During the hospital stay, 26 cats with respiratory signs (25.5%) had died or had been euthanised, while 76 cats (74.5%) were discharged alive and considered survivors. Short-term survival was compared between the various respiratory disease groups and it could be shown that cats in the neoplastic lower airway or pleural space disease group had a significantly higher risk of death than cats with UAD (*p* = 0.002), FLAD (*p* < 0.001) and CHF (*p* = 0.003). Cats that died had significantly higher SAA values at admission (median: 0.82 μg/mL, IQR: 0.11–6.49 μg/mL) compared to survivors (median: 0.59 μg/mL, IQR: 0.10–1.0 μg/mL) (*p* = 0.04; Figure 5). Hp values did not differ significantly between survivors and non-survivors. Long-term survival was not evaluated in the context of this study and therefore represents a limitation.

## Discussion

APPs are commonly used in veterinary medicine to tackle systemic inflammatory processes, but their diagnostic value in various feline respiratory diseases has not yet been investigated. This study examined the APPs SAA and Hp in cats with different respiratory diseases and investigated the association with clinical signs and short-term outcome. It was shown that cats with infectious lower airway or pleural space disease had higher, but not necessarily increased, levels of APPs in comparison to patients with other respiratory diseases. In contrast to other respiratory diseases, cats with FLAD had lower levels of APPs. In addition, most APP values were within the respective reference range despite presence of respiratory disease. It therefore does not appear possible to differentiate between various respiratory conditions on the basis of serum APP levels, and APP levels in the reference range cannot rule out ongoing respiratory disease.

Cats with CHF showed the shortest duration of clinical signs before presentation compared to cats with other respiratory diseases, which can be explained by the acute onset of the disease and is not a surprising result. In contrast, cats with FLAD had the longest history of disease, which emphasises the chronic nature of the disease. This finding corresponds with a previous study in cats evaluating duration of clinical signs in cats with respiratory and cardiac diseases (20). SAA concentrations of cats with CHF were shown to be weakly associated with the duration of clinical signs, which indicates higher SAA concentrations in cats with CHF presented with longer duration of clinical signs. A reason for this could be due to the fact that cats with CHF often present peracutely, and SAA may not yet have increased at that point. In comparison, cats with UAD had lower SAA levels, if the clinical signs were ongoing for a longer period of time. This could be due to the chronic nature of the disease, however SAA and Hp have been evaluated in chronic diseases in cats, such as CKD, inflammatory bowel disease and lymphoma, and investigations have shown increased levels compared to healthy cats (7, 21). Additionally, CHF is considered a non-inflammatory condition, and as observed in this study, APP values in most cats with CHF were within the reference range. This may also have influenced the analysis of a potential correlation with disease duration in cats with CHF. There was no other correlation between the duration of clinical signs before presentation and the level of APPs in this study. This is consistent with the result of a study in dogs with respiratory disease, which also found no effect of the duration of clinical signs on CRP concentration (18).

SAA was significantly higher in the infectious lower airway or pleural space disease group compared to cats with FLAD. A previous study has shown that cats with pneumonia had higher SAA values compared to healthy cats (14). However, a significant difference between the cats with infectious lower airway or pleural space disease and healthy cats could not be detected in the current study. One reason for the discrepancy in the results could be the heterogeneity of the infectious disease group, as well as the fact, that the type of pneumonia was not precisely defined in the previous study by Yuki and coworkers. A study investigating dogs showed that CRP, as the major APP in dogs, was significantly higher in bacterial pneumonia than in other respiratory diseases, such as chronic-inflammatory lower airway disease (chronic bronchitis and eosinophilic bronchopneumopathy), idiopathic pulmonary fibrosis, and CHF (18). Collectively, results suggest that increased SAA levels may be more indicative of infectious lower airway or pleural space disease as opposed to other respiratory diseases, especially FLAD. This could deliver helpful information when deciding on treatment in emergency settings, in case further diagnostics are considered too much of a risk due to instability of the patient and distinguishing between acute asthmatic attack and bacterial pneumonia can be challenging. However, it is important to keep in mind that a normal SAA value does not rule out an infectious cause. In addition, cats with higher SAA values in the infectious lower airway or pleural space disease group in this study were mainly cats with pyothorax. Most of the other cats with other types of infectious lower airway or pleural space disease showed values within the reference range, which means that not all types of infections necessarily lead to increased SAA values. In addition, cats with neoplastic lower airway disease and pleural effusion can also show high SAA levels, as seen in this study. Therefore, it appears overall difficult to distinguish between cats with infectious lower airway or pleural space disease and other respiratory diseases based on SAA and SAA should be used as supporting diagnostic information only.

Surprisingly, no significant difference was found when comparing SAA values between the other respiratory diseases or between the diseased and healthy cats. However, it should be noted that the median SAA levels of cats with infectious lower airway or pleural space disease were within the reference range and clinical relevance therefore appears to be low overall. However, a larger, prospective study investigating APPs in cats with respiratory diseases may expand upon these findings.

In this study, cats with FLAD showed significantly lower levels of Hp compared to cats with infectious or neoplastic lower airway or pleural space disease and UAD. Consequently, the presence of FLAD without an infectious component seems unlikely with elevated Hp values, but no statement can be made about other respiratory diseases based on the Hp values. Cats with UAD, neoplastic or infectious lower airway or pleural space disease and CHF showed significantly higher Hp levels compared to healthy cats. Despite the significant differences, the increased values were only mildly elevated and many cats showed levels within the normal range despite being diagnosed with respiratory disease. To the authors’ knowledge, no studies have investigated Hp levels in cats with respiratory disease and this study provides the first evidence. Overall, the interpretation of Hp values does not appear to provide significant added value in distinguishing between different feline respiratory diseases.

One possible reason why many cats diagnosed with respiratory disease showed APP values in the reference range could be the different time point of presentation and thus the time of measurement of the APPs. Major APPs, such as SAA, peak 24–28 h after stimulation, while moderate APPs, like Hp, peak 2–3 days after stimulation (22). The presentation and blood sampling of the cats at different stages of the disease could have influenced the results, as it must be assumed that many cats were not presented at the peak of the APPs due to the chronic character of the disease, or that the peak had not yet been reached in case of very acute presentation. However, this mimics the situation in real life daily practice and can therefore be used as a reference for clinical decision making. Repeated measurement of the APPs over the course of the disease would provide further important information about the behavior of the APPs in the respiratory diseases investigated. Based on the findings, the presence of a sterile chronic inflammatory airway disease such as FLAD appears less likely with elevated APP values.

Cats with lethargy and anorexia showed significantly higher SAA and Hp values compared to cats that did not show these respective clinical signs. An earlier study in cats has already shown that SAA is considered a marker for sepsis and can therefore indicate systemically severe inflammation (8). Lethargy and anorexia can be signs of a systemic inflammatory response and it is therefore not surprising that cats with reduced vitality due to systemic disease have elevated APP levels.

The APPs evaluated in this study were tested for correlation with short-term survival to discharge. Cats that died or were euthanised had significantly higher SAA levels compared to cats that survived. This result contradicts a previous investigation from Yuki and coworkers, which found that SAA in cats with various diseases, tested at admission, did not correlate with survival time (14). In comparison, another study failed to demonstrate that SAA and AGP could serve as a suitable prognostic marker in cats with lymphoma (5, 9). In the present study, Hp values were not significantly different between survivors and non-survivors. Previous studies investigating SAA and Hp in panleukopenic cats, showed significantly lower APP levels in cats that survived panleukopenia in comparison with non-survivors (23). Overall, it seems possible that higher SAA levels may lead to a poorer outcome in cats with respiratory disease, but caution should be taken when using APP levels as a prognostic marker based on current research. In this study, only survival to discharge was evaluated, and no long-term follow-up was performed. While APP levels may potentially provide indications regarding survival, they should not be used as basis for prognostic assessment or clinical decision-making at any time.

This study has several limitations including small sample size in each group as well as heterogenicity in disease groups. However, this is the first study evaluating various respiratory diseases in cats and provides motivation for larger future studies investigating APPs in cats with respiratory diseases. Serum samples were only obtained at the time of hospital admission, which was not necessarily the peak of the disease. While serial measurements at peak levels would be ideal to better reflect the dynamics of APPs, such an approach is not feasible in routine clinical practice and does not reflect conditions in everyday clinical work. Consequently, the analysis of APPs was limited to a single time point, which varied depending on the individual stage of disease at admission. Furthermore, only short-term follow-up data up to discharge were available. As a result, no conclusions can be drawn regarding the correlation between APP levels and long-term prognosis. In addition, data on the medical history and clinical data on the patients were extracted from the documented data and therefore obtained from various clinicians. However, since all patients were treated in the same hospital according to the principle of consensus, this does not appear to have had a significant impact on the results.

## Conclusion

Based on the results of this study, SAA and Hp, as important APPs in cats, cannot be used to clearly distinguish between different respiratory diseases. Higher SAA values could indicate the presence of infectious lower airway or pleural space disease compared to FLAD. Hp values do not provide any meaningful additional benefit in differentiating between various feline respiratory diseases. The results serve as preliminary data for future prospective studies investigating APPs in a larger number of cats with various respiratory diseases.

## Author’s note

Part of the study was presented as an oral presentation at the VCRS Symposium 2024 in Levi, Finland and as an oral presentation at the ECVIM Congress 2025, Maastricht, the Netherlands.

## Data Availability

The original contributions presented in the study are included in the article/supplementary material, further inquiries can be directed to the corresponding author.
